# Cardiovascular manifestations in severe and critical patients with COVID‐19

**DOI:** 10.1002/clc.23422

**Published:** 2020-07-15

**Authors:** Qingxing Chen, Lili Xu, Wenqing Zhu, Junbo Ge

**Affiliations:** ^1^ Department of Cardiology, Zhongshan Hospital Fudan University, Shanghai Institute of Cardiovascular Diseases Shanghai China

To the editor,

We would like to thank Kosuke Miki and Teruhiko Imamura for their thoughtful comments regarding our article titled “Cardiovascular Manifestations in Severe and Critical Patients with COVID‐19,” which was published last month in *Clinical Cardiology*.

In response to the concerns regarding the echocardiographic methodology of how to assess heart failure, we included strain assessment and tissue Doppler analyses during the assessment. We used ejection fraction (EF) as a measurement of left heart failure and tricuspid annular plane systolic excursion (TAPSE) as a measurement of right heart failure. We agreed that measurement of QRS duration could help assess the electrocardiographic myocardial injury, but we mainly evaluated the severity of myocardial injury by the concentration of cardiac troponin I (cTnI) and creatinine kinase‐MB isoenzyme (CK‐MB). Six patients (four severe and two critical cases) had a previous history of coronary artery disease. It is a pity that we did not apply angiography or computed tomography angiography (CTA) to survey the coronary artery for other patients, considering their severe condition. Time course of COVID‐19 was also our concern previously. But by the time we collected and analyzed the clinical data, the patients were all during treatment and we could not predict the final outcomes (live or death). So we focused our attention on analyses of risk factors for critical status at the time. And we did a follow‐up of these patients continuously. As for the third concern, we did not apply beta‐blocker in all these patients considering that beta‐blocker might exacerbate the lung condition.

